# Endobronchial Ultrasound Guided Transbronchial Needle Aspiration and PD-L1 Yields

**DOI:** 10.1007/s00408-024-00692-4

**Published:** 2024-04-18

**Authors:** Lara M. Khoury, Kristin N. Sheehan, William I. Mariencheck, Katherine A. Gershner, Matthew Maslonka, Angela G. Niehaus, Scott Isom, Christina R. Bellinger

**Affiliations:** 1 Department of Internal Medicine, Wake Forest University School of Medicine, Winston-Salem, NC USA; 2 Department of Pulmonary and Critical Care Medicine, Wake Forest University School of Medicine, Winston-Salem, NC USA; 3 Department of Pulmonary and Critical Care Medicine, Nebraska Pulmonary Specialties, Lincoln, NE USA; 4 Department of Pathology, Wake Forest University School of Medicine, Winston-Salem, NC USA; 5 Department of Biostatistics and Data Science, Wake Forest University School of Medicine, Winston-Salem, NC USA

**Keywords:** EBUS-TBNA, Bronchoscopy, PD-L1 yields, Molecular markers, Lung cancer

## Abstract

**Purpose:**

Immunotherapy is a leading approach for treating advanced non-small cell lung cancer (NSCLC) by targeting the PD-1/PD-L1 checkpoint signaling pathway, particularly in tumors expressing high levels of PD-L1 (Jug et al. in J Am Soc Cytopathol 9:485–493, 2020; Perrotta et al. in Chest 158: 1230–1239, 2020). Endobronchial ultrasound-guided transbronchial needle aspiration (EBUS-TBNA) is a minimally invasive method to obtain tissue for molecular studies, including PD-L1 analysis, in unresectable tumors (Genova et al. in Front Immunol 12: 799455, 2021; Wang et al. in Ann Oncol 29: 1417–1422, 2018). This study aimed to assess the adequacy of PD-L1 assessment in EBUS-TBNA cytology specimens.

**Methods:**

Data was collected retrospectively from patients who underwent EBUS-TBNA between 2017 and 2021 for suspected lung cancer biopsy. Samples positive for NSCLC were examined for PD-L1 expression. EBUS was performed by experienced practitioners, following institutional guidelines of a minimum of five aspirations from positively identified lesions. Sample adequacy for molecular testing was determined by the pathology department.

**Results:**

The analysis involved 387 NSCLC cases (149 squamous cell, 191 adenocarcinoma, 47 unspecified). Of the 263 EBUS-TBNA specimens tested for PD-L1, 237 (90.1%) were deemed adequate. While 84% adhered to the protocol, adherence did not yield better results. Significantly higher PD-L1 adequacy was observed in squamous cell carcinomas (93.2%) compared to adenocarcinoma (87.6%). The number of aspirations and sedation type did not correlate with PD-L1 adequacy in either cancer type, but lesion size and location had a significant impact in adenocarcinomas. Adenocarcinoma exhibited higher PD-L1 expression (68%) compared to squamous cell carcinoma (48%).

**Conclusion:**

EBUS-TBNA offers high yields for assessing immunotherapy markers like PD-L1, with satisfactory adequacy regardless of NSCLC subtype, lesion size, or location.

## Introduction

Lung cancer represents one of the leading causes of cancer-related deaths, but substantial advances have been made in enhancing the ways in which lung cancers are histologically identified and molecularly profiled. As a result, several targeted therapies have emerged as first line treatments [1, 2]. Immune checkpoint inhibitors (ICIs) target receptors such as programmed cell death protein 1 (PD-1) on T-cell lymphocytes or programmed death-ligand 1 (PD-L1) on tumor cells and optimize antitumor microenvironments via inhibition of checkpoint signaling pathways. ICIs promote host immune cell recognition of tumor cells and thereby indirectly downregulate tumor cell proliferation. For this reason, ICIs revolutionized cancer therapy in patients with advanced non-small cell lung cancers (NSCLC) and serve as frontline treatment for NSCLC with ≥ 50% PD-L1 expression. ICIs are also associated with improved overall survival outcomes in patients with advanced NSCLC when compared to those who receive cytotoxic chemotherapy.

In addition, the percentage of PD-L1 expression in tumor cells can prognosticate patient response to treatment, as higher levels of PD-L1 expression have been associated with better outcomes in those treated with ICIs [3]. Adequate tissue sampling is needed to optimize the yield of molecular markers that can be pharmaceutically targetable, such as PD-L1 [5]*.* A minimum of 100 viable tumor cells are needed to evaluate for PD-L1 expression, which may be limited by the small tissue samples obtained via EBUS-TBNA [4]. Therefore, in efforts to optimize personalized lung cancer therapies and identify indications for single modality ICI use, the methods in which tumor tissue samples are collected are important in determining personalized therapies.

Despite increased implementation of secondary prevention studies of asymptomatic lung cancers, approximately 70% of patients are diagnosed with NSCLC at advanced stages (stage III or IV) [6]. While surgical biopsies are reliable ways to obtain tumor tissue for molecular characterization, less invasive measures are typically preferred given decreased recovery time and incidence of post-procedural complications [7]. Endobronchial ultrasound-guided transbronchial needle aspiration (EBUS-TBNA) is a guideline-recommended (NCCN) minimally invasive approach used to diagnose, stage, and identify different subtypes of NSCLC [8]. EBUS-TBNA can also be used to obtain tissue for molecular studies in tumors that may not be amenable to resection or in patients whose medical comorbidities limit extensive intervention.

Several studies demonstrated that tumor tissue samples obtained via EBUS-TBNA are sufficient for PD-L1 testing, however, our study additionally investigates whether the subtype of NSCLC, size or location of tumor, and number of aspirations affect PD-L1 adequacy in EBUS-TBNA cytology specimens [1, 2, 7, 9].

## Methods

This is a retrospective study on patients who were suspected to have lung cancer on computed tomography (CT) or positron emission tomography (PET) and underwent EBUS-TBNA at Atrium Health Wake Forest Baptist between 2017 and 2021. The EBUS procedures were either performed under general anesthesia or moderate sedation using fentanyl and midazolam. A flexible Olympus EBUS bronchoscope attached to ultrasound convex probe transducer model and 21-gauge needles were used to sample endoscopically observed tumors. Our institution’s sampling protocol recommends a minimum of three fine needle aspirations (FNA) from a suspected benign lymph node, of which at least two are on slides for on-site cytology [10]. Once a suspected or confirmed (via on-site cytology) malignant lymph node is identified for additional sampling, our protocol is a minimum of three rinses into saline for pathology to perform immunohistochemical stains and molecular genetic testing.

The adequacy of the samples for molecular testing was determined by the pathology department and immunohistochemical (IHC) staining was performed for every patient case. Aspirated samples are expelled into a conical tube containing 5 cc of normal saline. The tube is then centrifuged at 2500–3000 rotations per minute for 5 min [11]. Following centrifugation, the supernatant is carefully decanted, and an equal amount of thrombin and plasma is added to facilitate clot formation, consolidating the cellular material into a cohesive cell block pellet [11]. This pellet is then transferred into a biopsy bag and subjected to processing procedures identical to those use for tissue biopsies, including immersion in 10% neutral-buffered formalin, embedding in paraffin wax, sectioning at 4 microns, and subsequent mounting onto positively charged slides [12]. Prior to immunohistochemical staining, hematoxylin and eosin (H&E) stained slides were evaluated for diagnosis and determination that sufficient tumor cells were present for accurate interpretation of PD-L1 immunohistochemistry. A minimum of 100 tumor cells are required for a single biopsy to evaluate PD-L1 expression [13]. Reflexively, all diagnoses of non-small cell lung carcinomas were stained for PD-L1 immunohistochemistry, using the commercial companion PD-L1 test (Dako 22C3 pharmDX).

Descriptive statistics, means and standard deviation, median and range, or counts and percentages, were calculated to describe the patient, lesion, and procedural characteristics. Exact tests were used to compare the distribution of lesion and procedural characteristics for adequate and insufficient samples for PDL-1, stratified by diagnosis. Results were considered significant when the p value was ≤ 0.05. The analysis for this paper was generated using SAS software, version 9.4 (SAS Institute Inc., Cary, NC).

## Results

Of 6223 bronchoscopies, 522 were EBUS-TBNA for adenopathy identified through prior radiographic imaging and NSCLC was identified in 387 patients (149 squamous cells, 191 adenocarcinomas, 47 not otherwise specified). Table 1 presents the patient demographics and characteristics of lesions. The mean age of the group was approximately 67.7 with a male to female ratio of 1.37, with 224 male and 163 women included in our study. The median number of sites sampled for each patient was 2.0. The most common locations sampled for molecular genetic testing were right paratracheal (24.8%), subcarinal (23.5%), and right hilar (20.7%) lymph nodes. Approximately 45.2% of the study group was diagnosed with stage IV lung cancer at the time of completing EBUS-TBNA.

**Table 1:** Patient Demographics and Lesion Characteristics

	All	Adenocarcinoma	NOS	Squamous Cell Carcinoma
Age, mean (sd)	67.7 (9.6)	67.6 (11.1)	66.9 (8.3)	68.0 (7.8)
Gender				
Female	163 (42.1%)	91 (47.6%)	17 (36.2%)	55 (36.9%)
Male	224 (57.9%)	100 (52.4%)	30 (63.8%)	94 (63.1%)
Number of sites sampled, median (min, max)	2.0 (1.0; 18.0)	2.0 (1.0; 18.0)	2.0 (1.0; 6.0)	3.0 (1.0; 15.0)
1–2	204 (52.7%)	108 (56.5%)	27 (57.4%)	69 (46.3%)
3 or more	183 (47.3%)	83 (43.5%)	20 (42.6%)	80 (53.7%)
Primary sampling location				
Parenchymal nodule / mass	43 (11.1%)	20 (10.5%)	6 (12.8%)	17 (11.4%)
High paratracheal (2R/2L)	2 (0.5%)	1 (0.5%)	0 (0.0%)	1 (0.7%)
L paratracheal (4L)	22 (5.7%)	9 (4.7%)	5 (10.6%)	8 (5.4%)
R paratracheal (4R)	96 (24.8%)	49 (25.7%)	13 (27.7%)	34 (22.8%)
Left hilum (10L/11L)	53 (13.7%)	24 (12.6%)	3 (6.4%)	26 (17.4%)
Right hilum (10R/11R)	80 (20.7%)	31 (16.2%)	12 (25.5%)	37 (24.8%)
Subcarinal (7)	91 (23.5%)	57 (29.8%)	8 (17.0%)	26 (17.4%)
Cancer Stage				
Stage 1	26 (6.7%)	14 (7.3%)	3 (6.4%)	9 (6.0%)
Stage 2	31 (8.0%)	11 (5.8%)	3 (6.4%)	17 (11.4%)
Stage IIIA	70 (18.1%)	30 (15.7%)	8 (17.0%)	32 (21.5%)
Stage IIIB	85 (22.0%)	33 (17.3%)	8 (17.0%)	44 (29.5%)
Stage IV	175 (45.2%)	103 (53.9%)	25 (53.2%)	47 (31.5%)

Table 2 demonstrates the characteristics of the procedure. More patients (*n* = 298, 77.0%) received general anesthesia compared to 89 patients (23.0%) received moderate sedation. The mean total procedure time was longer among patients who received general anesthesia (53.0 min) than those who received moderate sedation (37.8 min). The complication rate was 4.1%, consisting of arrhythmia (*n* = 2, 0.5%), hypoxia requiring admission (*n* = 3, 0.8%), overall intolerance of procedure (*n* = 5, 1.3%), transient bronchospasm (*n* = 3, 0.8%), and other not specified complications (*n* = 3, 0.8%).

**Table 2:** Procedural Characteristics

General Anesthesia (n,%)	298 (77.0%)
Procedure time, in minutes, mean (SD)	53.0 (21.5)
Moderate Sedation (n,%)	89 (23.0%)
Procedure time, in minutes, mean (SD)	37.8 (12.8)
Complications (n,%)	
None	371 (95.9%)
Arrhythmia	2 (0.5%)
Hypoxia requiring admission	3 (0.8%)
Poor overall tolerance of procedure	5 (1.3%)
Transient bronchospasm or desaturation	3 (0.8%)
Other	3 (0.8%)

The PD-L1 yields identified in both adenocarcinoma and squamous cell carcinoma cases are described in Table 3. A total of 263 EBUS-TBNA specimens were evaluated for adequacy with 110 (93.2%) of the squamous cell carcinoma samples and 127 (87.6%) of the adenocarcinoma samples deemed adequate for PD-L1 testing. The remaining 124 EBUS-TBNA specimens in which NSCLC was identified were not evaluated for adequacy for reasons unclear in chart review. There was no statistical difference between insufficient and adequate yields collected in squamous cell carcinoma cases based on the primary location the tissue was collected, size of tumor, number of aspirations, or type of sedation. However, statistical differences were appreciated between the insufficient and adequate yields collected in adenocarcinoma cases stratified by location (*p* = 0.03) with greater yields in subcarinal samples, and size (*p* < 0.01) with larger yields in tumors measuring > 3 cm, but not by number of aspirations (*p* = 0. 3473) or type of sedation (*p* = 0.3269).

**Table 3:** Yields PDL-1

	Squamous Cell Carcinoma			Adenocarcinoma			
	Insufficient	Adequate	p-value	Insufficient		Adequate	p-value
N	8	110		18		127	
Primary location			0.1642				0.0273
Parenchymal nodule / mass	0 (0.0%)	15 (100%)		3 (16.7%)		15 (83.3%)	
Paratracheal	3 (10.7%)	25 (89.3%)		5 (11.6%)		38 (88.4%)	
Hilum	4 (7.4%)	50 (92.6%)		7 (15.2%)		39 (84.8%)	
Subcarinal (7)	1 (4.8%)	20 (95.2%)		3 (7.9%)		35 (92.1%)	
Size			0.4578				0.0012
< 1 cm	0 (0.0%)	2 (100%)		1 (9.1%)		10 (90.9%)	
1 to < 2 cm	3 (12.5%)	21 (87.5%)		5 (14.3%)		30 (85.7%)	
2 to < 3 cm	2 (6.7%)	28 (93.3%)		9 (20.9%)		34 (79.1%)	
≥ 3 cm	3 (4.8%)	59 (95.2%)		3 (5.4%)		53 (94.6%)	
Number of aspirations			0.7229				0.3473
1–4	1 (4.8%)	20 (95.2%)		2 (14.3%)		12 (85.7%)	
5–7	3 (9.1%)	30 (90.9%)		2 (6.1%)		31 (93.9%)	
8–10	3 (6.7%)	42 (93.3%)		7 (10.0%)		63 (90.0%)	
> 10	1 (5.3%)	18 (94.7%)		7 (25.0%)		21 (75.0%)	
Sedation Type			0.5846				0.3269
General Anesthesia	7 (7.4%)	87 (92.6%)		13 (12.0%)		96 (88.0%)	
Moderate Sedation	1 (4.2%)	23 (95.8%)		5 (13.9%)		31 (86.1%)	

Table 4 demonstrates the percentage of PD-L1 expression in FNA sufficient yields in both squamous cell and adenocarcinoma cases. Most of the adequate cell yields in squamous cell carcinoma cases had no PD-L1 expression (PD-L1 < 1%) (51.8%), whereas 27.3% had low expression (PD-L1 1–49%) and 20.9% had high PD-L1 expression (PD-L1 ≥ 50%) (*p* = 0.003). Expression of PD-L1 was found to be higher in adenocarcinoma (67.7%) compared to SCC (48.2%). In adenocarcinoma samples 29.1% had no PD-L1 expression, 37.8% had low PD-L1 expression, and 29.9% had high PD-L1 expression.

**Table 4:** Percentage of PDL-1 Expression in Sufficient FNAs

	Squamous Cell Carcinoma	Adenocarcinoma
< 1%	57 (51.8%)	37 (29.1%)
1- 49%	30 (27.3%)	48 (37.8%)
≥ 50%	23 (20.9%)	38 (29.9%)
Unknown	0	4

High Expression: TPS ≥ 50%. Low Expression: TPS 1–49%. No expression: TPS < 1%

## Discussion

Molecular profiling revolutionized NSCLC management by helping personalize therapies with identification of targetable biomarkers and predicting the efficacy of ICIs for the treatment of advanced NSCLC [14]. Given that most patients are diagnosed with NSCLC at advanced stages rendering them medically vulnerable, minimally invasive measures are needed to avoid incidence of post-procedural complications [6, 15]. Our study demonstrated that EBUS-TBNA offers a minimally invasive approach to obtaining adequate diagnostic yields for molecular profiling from sampled tissue specimens regardless of subtype of NSCLC, lesion size, or location of lesion.

Of the 263 EBUS-TBNA specimens that were evaluated for adequacy, 237 (90.1%) EBUS-TBNA were adequate for PD-L1 testing, which is consistent with several previous studies that reported 80–100% adequate yields obtained by EBUS-TBNA for PD-L1 IHC testing [7, 16–20]. Prior studies investigated the differences in PD-L1 expression in tissue samples collected using bronchoscopy (TBB or TBNA) compared to those collected using CT vs US guided needle core biopsies. Although larger samples were noted when tumor tissue was collected via CT and US guided needle core biopsies, all yields were adequate regardless of sampling modality [18, 19]. Despite lower tumor cell counts found in EBUS-TBNA samples in comparison to biopsies obtained through more invasive measures, EBUS-TBNA still provided adequate yields for sufficient tumor proportion scoring (TPS) [21]. Heymann, J.J. et al., demonstrate comparable adequate yields obtained through EBUS-TBNA (90%), core needle biopsy (96%), and resected biopsy (99%) [19]. There is paucity in studies comparing tumor size and location to adequate yields obtained via EBUS-TBNA, however, our study additionally demonstrated that PD-L1 yields were significantly higher in specimens that were obtained from tumors measuring > 3 cm and biopsies taken from subcarinal lymph nodes in adenocarcinoma cases.

Like other studies that demonstrated variability in TPS among subtypes of cancers, our results revealed that PD-L1 expression was found to be significantly higher (p < 0.01) in adenocarcinoma (67.7%) compared to SCC (48.2%). However, those other studies additionally appreciated higher TPS in advanced stages of adenocarcinoma than those in the early stages of adenocarcinoma [18, 22]. Although we did not evaluate which stages were related to the adequate and highest PD-L1 expressing yields in adenocarcinoma cases, 86.9% (166) of the 191 identified adenocarcinoma cases were identified at advanced stages (III and IV).

One of the strengths of our study is that we tested a range of percentages for PD-L1 expression. This is important because when comparing the adequacy of PD-L1 yields between EBUS-TBNA obtained specimens and surgically resected tumor biopsies at two separate cutoffs (≥ 1% and ≥ 50%), a significant decrease in the positive predictive value and sensitivities associated with a PD-L1 expression cutoff of ≥ 50% in EBUS-TBNA samples were observed. Therefore, higher cutoffs (≥ 50%) can poorly estimate PD-L1 status and result in false negatives, which are commonly noted in samples that have low cellularity, such as those obtained via EBUS-TBNA, thereby necessitating a range of percentages [21]. Moreover, our study consisted of one of the largest sample sizes (*n* = 237) of adequate EBUS-TBNA specimens to date.

The limitations to our study include that it is a single center retrospective observational study that is prone to biases with dependence on prior documentation. Although tumor heterogeneity in PD-L1 expression is an uncontrollable variable, we acknowledge that there are limitations in the accuracy of reported TPS between samples obtained from different tumor sites within each patient [17, 23]. This study additionally did not specify which patients already received cancer treatment prior to tissue sampling, which could affect PD-L1 expression found in samples [24]. A better understanding of how the measurement of PD-L1 expression in EBUS-TBNA specimens affected the subsequent design of personalized cancer therapies would have been helpful and could have been compared to a previous study that demonstrated that patients whose EBUS-TBNA samples reflected higher PD-L1 expression had near complete treatment response after ICI initiation [16].

## Conclusion

EBUS-TBNA is a minimally invasive approach associated with a low incidence of post-procedure complications. It offers adequate sampling of tumor tissue needed for molecular profiling, which is a critical component of personal cancer therapies. Sufficient yields for PD-L1 testing can be collected via EBUS-TBNA regardless of number of aspirations, size or location of lesion, or subtype of NSCLC.

## Data Availability

All data generated or analyzed during this study are included in this article and its supplementary material files. Further inquiries can be directed to the corresponding author.
